# Experimental Evolution of Pathogenic *Candida* spp.: Insights into Adaptive Processes and Evolutionary Dynamics

**DOI:** 10.3390/microorganisms14020273

**Published:** 2026-01-24

**Authors:** Gonçalo Sousa, Inês Correia, Ana Rita Bezerra

**Affiliations:** Institute of Biomedicine—iBiMED, Department of Medical Sciences, University of Aveiro, 3810-193 Aveiro, Portugal; goncalofsousa@ua.pt (G.S.); inescorreia@ua.pt (I.C.)

**Keywords:** fungal pathogens, experimental evolution, evolutionary genomics, host adaptation, antifungal resistance

## Abstract

Among *Candida* species, several are major opportunistic fungal pathogens capable of causing a wide spectrum of infections, ranging from superficial mucosal conditions to severe systemic diseases. Their success as human pathogens is largely due to their ability to rapidly adapt to diverse host environments and develop resistance to antifungal agents. Experimental evolution provides a powerful framework for understanding these adaptive processes by observing evolutionary change in real-time. Although most studies rely on in vitro systems and a limited set of *Candida* species, there is strong evidence that genome plasticity, including aneuploidy, loss of heterozygosity, and copy number variation, plays a central role in driving rapid adaptation. Experimental evolution has also been applied to study the dynamics of antifungal resistance, particularly to azoles, although relatively fewer studies have explored resistance to echinocandins and polyenes. This review summarizes current knowledge on experimental evolution in pathogenic *Candida* species, with a focus on genome plasticity, adaptation to host-imposed stress, and particularly on the emergence of antifungal resistance. It also identifies critical research gaps, including the need for broader species coverage, investigation of underexplored antifungal classes, and evaluation of combined therapies. A deeper understanding of these dynamics is essential to improve antifungal strategies and counter the growing threat of drug-resistant *Candida* spp. infections.

## 1. Introduction

Experimental evolution (EE) involves the study of evolutionary change in populations subjected to defined environmental, demographic, genetic, social, and other constraints imposed under controlled laboratory or natural conditions [1]. Over time, EE has been adapted and refined across multiple disciplines, leading to the development of distinct methodologies suited to specific research questions and model systems [2,3,4,5,6]. Its growing importance is highlighted by the sharp increase in EE studies over the past decade, reinforcing the relevance of reviewing its applications.

Microbial EE typically uses well-controlled laboratory populations combined with molecular and genomic tools to investigate mechanisms of adaptation, validate theoretical predictions, and discover novel evolutionary outcomes [7,8,9]. In the context of human health, EE has been instrumental in studying the development of antimicrobial resistance, particularly among invasive fungal pathogens [10,11].

Fungal infections represent a major clinical concern, particularly in immunocompromised or critically ill patients [12,13,14]. The range of potential pathogens responsible for these infections is expanding, with *Candida* spp., *Cryptococcus neoformans*, *Pneumocystis jirovecii*, and *Aspergillus* spp. being the most prevalent [15]. *Candida* species can cause invasive fungal infections, particularly bloodstream infections, which are associated with high morbidity and mortality rates [16]. While *C. albicans* remains the most commonly isolated species, non-albicans *Candida* (NAC) species, such as *C. glabrata* (reclassified as *Nakaseomyces glabratus* [17]), *C. krusei* (reclassified as *Pichia kudriavzevii* [18]), *C. parapsilosis*, and *C. tropicalis*, are increasingly prevalent and often show resistance to azole antifungals, especially fluconazole [19]. More recently, the emergence of the multidrug-resistant yeast *C. auris* (reclassified as *Candidozyma auris* [19]) has further underscored the urgent need to understand how antifungal resistance evolves [20,21,22].

Previous reviews of EE assays have highlighted their value for investigating antifungal drug resistance in pathogenic fungi, particularly via drug-driven selection experiments that reconstruct resistance evolution under controlled conditions. However, most studies have focused exclusively on resistance phenotypes, whereas the use of EE to study other fungal traits, such as genome plasticity and the response to host-related stresses, has not yet been comprehensively synthesized.

In this review, we focus on EE studies in pathogenic *Candida* species and aim at capturing the multiple evolutionary dimensions shaping fungal pathogens beyond antifungal exposure. We synthesize current knowledge on three major themes: (i) evolutionary dynamics of genome plasticity, (ii) adaptation to host-imposed stressors, and (iii) the evolution of antifungal drug resistance. By integrating these areas, we aim to provide a unified view of how EE can help elucidate clinically relevant evolutionary processes and guide the development of more effective strategies to combat antifungal resistance.

## 2. Literature Search Strategy

A comprehensive literature search was conducted across the electronic database PubMed (PubMed.gov from the National Library of Medicine). The search covered publications from 1990 to November 2025. Keyword combinations included “experimental evolution”, “in vitro experimental evolution”, “in vivo experimental evolution”, “antifungal drug resistance”, “fungal pathogens”, “*Candida* species”, “*Candida albicans*”, “Non-*albicans Candida*”, “host-imposed stress”, and “genomic flexibility”. After the initial retrieval, titles and abstracts were screened to identify original research and key reviews examining the potential of experimental evolution to study fungal adaptation. Papers aligned with the aims of this review were subsequently selected for in-depth analysis and synthesis. PubMed was chosen as the primary database because of its strong coverage of biomedical, microbiological, and clinical literature relevant to pathogenic fungi. Nevertheless, we acknowledge that some experimental evolution studies may also be indexed in other databases.

## 3. Experimental Evolution as a Tool to Study Pathogenic *Candida* spp.

**Figure 1:**
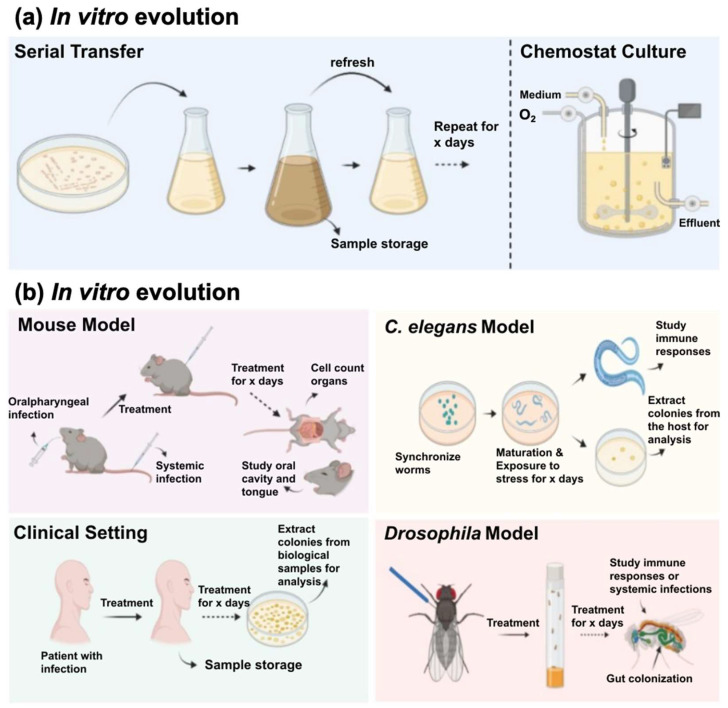
Overview of experimental evolution approaches. (**a**) In vitro evolution includes two main approaches: serial transfer, where microbial populations are repeatedly propagated in fresh medium, promoting adaptation (e.g., antifungals, macrophages, H_2_O_2_) across successive growth cycles, and chemostat culture, a continuous system in which fresh medium is supplied and culture is removed at a constant rate, maintaining a steady state and allowing adaptation under stable selective pressures (e.g., antifungals). (**b**) In vivo evolution relies on different host models: the mouse model, often established through antimicrobial treatment of pathogens in immunocompetent or immunocompromised mice to study infection dynamics and immune adaptation; the *Caenorhabditis elegans* model, where nematodes are infected on agar plates or liquid culture to monitor pathogen adaptation in a simple host; the *Drosophila* model, typically involving infection through pricking or feeding assays to explore genetic determinants of host–pathogen interactions; and the human host, where evolutionary dynamics are observed directly during clinical infections, for instance through longitudinal sampling of isolates from patients under antimicrobial treatment.

EE has become a powerful tool for investigating adaptive processes in fungal pathogens. While EE has been widely applied to diverse fungal genera (e.g., *Cryptococcus*, *Aspergillus*, *Saccharomyces*), this review deliberately focuses on pathogenic *Candida* species as they combine high clinical relevance, genetic tractability, and a substantial body of in vivo and in vitro EE studies directly linked to antifungal resistance and host adaptation. *Candida* species are especially suitable for EE due to their relatively short generation times, large population sizes, and well-developed molecular and genomic toolkits. In the study of pathogenic *Candida* spp., understanding adaptation requires integrating both in vitro EE and in vivo evolution approaches (Figure 1).

EE techniques such as “laboratory natural selection” and “laboratory culling” are frequently used to simulate environmental stressors without explicitly selecting for specific traits. In “laboratory natural selection,” researchers alter environmental parameters, such as temperature, nutrient availability, or antifungal presence, while maintaining an unaltered control population for comparison [1]. In contrast, “laboratory culling” involves applying lethal or sublethal stress to a population and allowing only the survivors to seed the next generation, thereby mimicking strong selection pressure [1]. These experimental designs help reveal the diversity of adaptive strategies that pathogenic *Candida* species can evolve, including changes in drug susceptibility, stress tolerance, or virulence factors expression.

However, one major limitation of laboratory-based EE is its ecological relevance. The controlled and simplified conditions used in vitro often fail to replicate the complexity of host environments, where *Candida* encounters dynamic pressures such as immune responses, microbiota interactions, fluctuating nutrient availability, and tissue-specific conditions. These limitations can restrict the applicability of in vitro findings to clinical or natural scenarios. Furthermore, the relatively short timescales typical of laboratory studies might not capture long-term evolutionary dynamics that are relevant to chronic or recurrent infections [1].

To address these limitations, in vivo evolution studies have been conducted using animal models (e.g., mice, nematodes and insects) or through the longitudinal analysis of clinical isolates (clinical cohorts). These studies are particularly valuable for understanding how *Candida* adapts to immune responses, anatomical niches, and therapeutic interventions within living hosts [23]. In vivo evolution provides essential insights into the development of antifungal resistance, persistence mechanisms, and virulence evolution. Nevertheless, ethical and logistical constraints, especially in studies involving humans or animals, limit the frequency, reproducibility, and scope of such investigations. Additionally, the complexity of host environments makes it difficult to isolate specific selective forces, and the lack of environmental control introduces confounding variables that can complicate result interpretation and make experimental replication challenging.

The integration of EE with next-generation sequencing has further advanced the field by enabling the detailed characterization of genomic responses to selection [5]. For example, comparative studies have shown that *Candida albicans* populations evolved in vivo (e.g., within oral host niches) exhibit slower but more diverse genomic and phenotypic changes than those evolved under in vitro conditions [24]. Isolates from host environments can generate highly divergent isolates, with mutation and diversity rates up to two orders of magnitude greater than isolates from laboratory setting [25].

Despite their differences, both in vitro and in vivo EE approaches are essential for understanding the evolutionary dynamics of pathogenic *Candida* spp. and for developing strategies to manage the high burden of fungal infections. In vitro EE is particularly well-suited for dissecting specific mechanisms, such as the emergence of antifungal resistance biomarkers, while in vivo evolution is indispensable for exploring complex host–pathogen interactions in realistic biological settings.

## 4. Evolutionary Dynamics of Genome Plasticity in Pathogenic *Candida* Species

Genetic variation is a central feature of pathogenic *Candida* species biology, playing a critical role in their ability to adapt to diverse environments, evade host immune responses, and develop resistance to antifungal agents. *C. albicans*, in particular, exhibits extensive genome plasticity, driven by genetic mutations, chromosomal rearrangements, loss of heterozygosity (LOH), copy number variation (CNV), and variation in ploidy. EE studies have been instrumental in uncovering the extent and mechanisms of this plasticity under both stress-free and selective conditions [26]. Table 1 summarizes key studies on these genomic changes and their adaptive outcomes, revealing that most research has focused on *C. albicans* while non-*albicans Candida* (NAC) species remain largely unexplored.

Ploidy shifts between haploid, diploid, and/or polyploid states occur frequently in *C. albicans* and are mediated by both sexual and asexual processes. These transitions introduce genetic diversity within a cell population and enable rapid adaptation [26,27]. Hickman and colleagues studied the stability of *C. albicans* tetraploid and aneuploid isolates by performing in vitro EE over 28 days in nutrient-rich medium. They tracked ploidy changes using a combination of flow cytometry and restriction-site-associated DNA sequencing [27]. Their findings showed that isolates have a strong tendency towards euploidy, with diploids being the most stable state. Polyploid and aneuploid states were fundamentally unstable, and isolates that remained polyploid showed increased variability, providing a genetic basis for selection [27]. Gerstein et al. extended this analysis by evolving *C. albicans* strains with different initial ploidy states (haploid, diploid, polyploid) across four media—synthetic defined complete medium (SDC), minimal medium (MM), phosphorus-depletion medium (Pdep), and nitrogen-depletion medium (Ndep)—over approximately 140 generations [28]. They found that, in the four different media, 96% of diploid lines remained diploid, while haploid and polyploid populations were more prone to ploidy shifts. The frequency and direction of ploidy changes depended on the growth environment: while SDC favoured haploid-to-diploid transitions, MM and Pdep impaired them. Genome size was also influenced by environmental conditions, with larger genomes observed in SDC and Ndep [28]. These studies emphasize that ploidy instability generates transient genetic diversity that can be shaped by selection and environmental stress.

EE has also allowed quantification of spontaneous mutation rates and genome rearrangement events. Ene et al. conducted a long-term EE study in *C. albicans* strain SC5314 and clinical isolates over 80 days and estimated a de novo base substitution rate of 1.17 × 10^−10^ per base pair per generation, and an LOH rate of 1.61 × 10^−10^ per base pair per generation via gene conversion [29]. Other study found that CNV rates ranged from ~1.3 × 10^−6^ to 1.5 × 10^−6^ per gene per cell division [30]. These mutations were disproportionately located in repeat-rich and subtelomeric regions, intergenic sequences, and genes encoding GPI-linked cell wall proteins. Notably, these rates were observed under standard laboratory conditions; mutation rates are likely increased in vivo due to the activation of stress responses during host colonization and immune evasion [29,30].

Repetitive elements, such as transposons, tandem repeats, and long inverted sequences, contribute significantly to the genomic plasticity of *C. albicans*. Subtelomeric regions exhibit high variability, often containing lineage-specific genes with roles in stress response and pathogenesis. In the *C. albicans* SC5314 reference strain, subtelomeric regions harbour approximately 50 protein-coding genes, many linked to biofilm formation, hyphal growth, and metabolism [31]. Anderson and colleagues evolved this strain over 4000 generations in nutrient-rich media to investigate subtelomeric genome evolution. They found that these regions accumulated high levels of Single-Nucleotide Polymorphisms (SNPs), Insertion and Deletions (INDELs), and CNVs, driven by ectopic recombination and point mutations [32]. About 25% of the rapidly evolving subtelomeric genes belonged to the *TLO* family, which regulates virulence-associated traits including stress resistance and filamentation [31].

Todd and colleagues also studied genome plasticity driven by long repeat sequences in *C. albicans* strains evolved in vitro [33]. Their work showed that an isolate with a long-inverted repeat within the centromere of chromosome IV can generate an isochromosome 4L, which remains stable for at least 300 generations and enhances fitness in the presence of fluconazole. This structure was associated with LOH breakpoints on chromosome 4L. Another isolate featured a CNV breakpoint on chromosome 3L, associated with intergenic sequences [33]. These studies collectively emphasize the significance of repetitive elements and long repeat sequences in shaping the genomic architecture and plasticity of pathogenic *Candida* species.

Most EE studies to date have focused on *C. albicans*, leaving a gap in our understanding of genome plasticity in NAC species such as *C. glabrata*, *C. tropicalis*, and *C. auris*. Given their increasing clinical importance and divergent evolutionary strategies (e.g., reduced recombination in *C. glabrata*), further comparative evolution studies are needed to assess whether similar plasticity mechanisms operate in these species.

From a clinical and experimental perspective, these findings emphasize that genome plasticity is a central driver of adaptation, virulence, and potential antifungal resistance in *Candida* spp. Environmental conditions shape the pace and nature of these genomic changes, and EE is uniquely suited to dissect these dynamics. Understanding how genome instability contributes to antifungal resistance and virulence can inform therapeutic development and long-term infection management strategies.

**Table 1:** Summary of relevant EE studies on genome plasticity and adaptive phenotypes in *Candida albicans*.

Study	Condition	Genomic Change	Adaptive Phenotype
Hickman et al. [27]	In vitro, nutrient-rich medium (28 days)	Ploidy shifts; tendency of polyploids/aneuploids toward euploidy (diploid)	Recovery of the stable diploid state; polyploidy provides genetic variability for selection
Gerstein et al. [28]	Multiple media (SDC, MM, Pdep, Ndep) over 140 generations	Haploid-to-diploid transitions; changes in genome size	Environment-specific stability (SDC favours diploidy; MM and Pdep impair transitions)
Ene et al. [29]	Long-term (80 days) standard laboratory conditions	De novo base substitutions and LOH	Baseline quantification of mutation rates (1.17 × 10^−10^ bp/gen) for evolutionary potential
Todd et al. [30]	Standard laboratory conditions	CNV in repeat-rich and subtelomeric regions	Diversification of GPI-linked cell wall proteins; preparation for host colonization
Anderson et al. [32]	Nutrient-rich media (4000 generations)	SNPs, INDELs, and CNVs in subtelomeric regions (specifically *TLO* genes)	Regulation of virulence-associated traits like stress resistance and filamentation
Todd et al. [33]	In vitro evolution	Formation of isochromosome 4L; CNV breakpoints at long-inverted repeats	Enhanced fitness/resistance in the presence of fluconazole (antifungals)

Abbreviations: SNP, Single-Nucleotide Polymorphism; INDEL, Insertion and Deletion; CNV, Copy Number Variation; LOH, Loss of Heterozygosity.

## 5. Evolutionary Adaptation of *Candida* to Host-Imposed Stressors

### 5.1. Candida albicans

Pathogenic *Candida* species exhibit remarkable adaptability to various environments, allowing them to thrive in diverse ecological niches. This adaptability is crucial for their persistence, pathogenesis, and capacity to colonize and infect both host-associated and external environments. Within the human host, pathogenic *Candida* spp. colonizes mucosal surfaces such as the gastrointestinal tract, oral cavity, and reproductive organs, where they encounter dynamic stressors including pH fluctuations, immune surveillance, nutrient competition, and interactions with commensal microbes [34].

To investigate host-associated adaptation, EE studies using in vivo models have been instrumental. Forche and colleagues demonstrated that *C. albicans* undergoes frequent LOH events during serial passage in mouse models, along with karyotypic changes and phenotypic diversification [24,35]. LOH events, arising from mitotic recombination, were distributed across various genomic loci and often co-occurred with phenotypic shifts, such as hypha formation, indicating that *C. albicans* adapts through diverse genomic routes in response to host-induced selection. Further, in an oral mouse model, Forche and colleagues showed that evolved isolates displayed a variety of genomic changes in all chromosomes, including aneuploidies in small chromosomes and LOH in larger chromosomes, enabling a possible rapid adaptation to the diversity of stress environments it encounters inside the host [25].

Studies using the nematode *Caenorhabditis elegans* as an infection model revealed similar trends. Smith et al. explored the influence of the genetic background and ploidy on *C. albicans* genome stability, finding that host-induced LOH events and genome size changes varied significantly depending on the strain and ploidy state. Tetraploid strains were particularly unstable, often undergoing genome reductions associated with attenuated virulence, while diploid strains showed smaller genomic shifts but increased virulence [36]. In a subsequent study, Smith and colleagues demonstrated that host immune competence further modulates genome instability. When *C. albicans* was passage through immunocompromised nematodes lacking key immune effectors such as reactive oxygen species (ROS) or antimicrobial peptides (AMPs), the frequency of LOH and aneuploidies decreased, and the evolved isolates exhibited lower virulence [37]. The same research group published a follow-up study, demonstrating that tetraploid *C. albicans* strains evolved increased virulence while undergoing extensive genome size reductions, ultimately approaching a diploid state. This rapid evolution of virulence and genome size occurred in hosts irrespective of immune status; however, in immunocompetent hosts, the process followed complex temporal dynamics, characterized by an initial rapid increase in virulence followed by its subsequent decline [38]. These findings emphasize the selective role of host immunity in shaping fungal genome dynamics and virulence evolution.

Furthermore, Ene and colleagues demonstrated that *C. albicans* undergoes elevated base-substitution rates during gastrointestinal colonization in mice [29]. Large-scale chromosomal changes were relatively rare, although chromosome VII’s trisomy frequently emerged during passaging in this model and was associated with increased fitness for this niche. Multiple chromosomal features impacted mutational patterns, with mutation rates elevated in repetitive regions, subtelomeric regions, and in gene families encoding cell surface proteins involved in host adhesion.

Other studies also validated the fact that genomes show biased patterns of mutations suggestive of extensive purifying selection during evolution [29]. Tso et al. exposed *C. albicans* to evolutionary pressure through repeated passages in antibiotic-treated mice devoid of gut bacteria. This process led to selection for low-virulence phenotypes with mutations near the *FLO8* gene, which lost hyphal formation capacity but still stimulated immune responses. However, in microbiota-replete hosts, only hyphal (virulent) forms persisted, suggesting the microbiome acts as an additional evolutionary filter promoting pathogenic traits [39]. A recent study employing a *Drosophila melanogaster* infection model demonstrated its utility for investigating *C. albicans* pathogenesis and assessing antifungal interventions [40]. *C. albicans* 4372, a clinical isolate obtained from a pleural fluid sample, displayed enhanced virulence by eliciting a dysregulated innate immune response, resulting in rapid host mortality despite effective control of fungal burden. This phenomenon was characterized by accelerated and amplified production of AMPs and other immune effectors. These findings highlight that strain-specific virulence can arise not only from direct pathogenic mechanisms but also from the induction of immunocompromised host responses [40].

*C. albicans* also adapts by altering their morphology, switching between yeast and filamentous forms, modifying their cell wall composition, and expressing virulence factors to evade host defences and establish infection. To explore the relevance of the filamentation phenotype, Wartenberg et al. undertook in vitro evolution of a non-filamentous *C. albicans cph1/efg1* double mutant in the presence of macrophages [41]. Remarkably, after 42 passages, the strain regained the ability to filament due to a compensatory missense mutation (R352Q) in *SSN3*, a gene encoding a Cdk8 module component of the Mediator complex involved in the co-regulation of transcription [42]. This mutation bypassed the need for *CPH1* and *EFG1*, restoring filamentation and upregulating core filamentation genes [41].

Collectively, these studies describe how genomic alterations, such as LOH, aneuploidy and point mutations influence adaptability to the host immune system and affect its response to the pathogen. However, further EE studies are needed to explore other virulence traits, such as adhesion and biofilm formation.

### 5.2. Candida glabrata

Although *C. albicans* is the most used fungal pathogen in EE studies, other *Candida* pathogens also demonstrate adaptive flexibility. Brunke et al. serially passaged *C. glabrata* with macrophages over six months. This evolution resulted in a striking alteration in fungal morphology. The growth form changed from typical spherical yeasts to pseudohyphae-like structures, a phenotype which was stable over several generations without any selective pressure [43]. This morphology was accompanied by changes in cell wall architecture, faster escape from macrophages, increased macrophage damage, and enhanced virulence. This phenotype was linked to an N556K substitution caused by a genetic point mutation in *CHS2*, a gene encoding a chitin synthase, illustrating how single-nucleotide changes can significantly impact pathogenic traits [43].

Adaptation to oxidative stress is another hallmark of *Candida* survival within the host. Huang and colleagues performed short-term adaptive evolution of *C. glabrata* under increasing hydrogen peroxide concentrations, from 80 mM to 350 mM, over ~180 generations [44]. Through genome resequencing and transcriptomic profiling, key resistance genes including *CgCTH2* (CAGL0E01243g) and *CgMGA2* (CAGL0F06831g) were identified. These genes were implicated in NADPH regeneration, cell wall remodelling, membrane composition, and global transcriptional regulation. Evolved strains exhibited enhanced detoxification and growth in oxidative environments, underscoring the multi-layered genetic and functional adaptations *C. glabrata* employs to counter host-derived ROS.

Overall, pathogenic *Candida* species, particularly *C. albicans*, display remarkable genomic plasticity during host colonization and infection, including LOH, aneuploidy, base-substitution mutations, and compensatory changes that restore lost traits. These genomic changes are tightly linked to phenotypic adaptation, such as altered virulence, filamentation, and stress resistance, and are strongly shaped by host-imposed pressures including immune effectors, nutrient availability, and microbiota composition. Evolutionary trajectories are influenced by strain background and ploidy, with tetraploid strains often evolving more rapidly but undergoing extensive genome instability, while diploid strains show more gradual genomic shifts. Collectively, these studies highlight that *Candida* adaptation is context-dependent, dynamic, and capable of generating diverse strategies to persist and thrive within the host.

Clinically, these findings underscore the importance of understanding host-driven adaptation for predicting virulence and resistance emergence, particularly in immunocompromised patients or those receiving microbiota-disrupting treatments. From an experimental perspective, in vivo and in vitro EE models provide critical frameworks for dissecting evolutionary mechanisms, allowing researchers to identify generalizable principles, such as the interplay between genome instability, compensatory mutations, and phenotypic outcomes. Integrating genomic, phenotypic, and host-context data can reveal potential intervention targets and guide the design of future studies aimed at anticipating the adaptive potential of pathogenic *Candida* species in clinical settings.

## 6. Evolution of Antifungal Drug Resistance in *Candida* Species

There are five major classes of antifungal agents, polyenes, azoles, echinocandins, allylamines, and antimetabolites; however, only three (polyenes, azoles, and echinocandins) are routinely used for systemic treatment of invasive candidiasis. Allylamines and antimetabolites are primarily used for superficial infections or for specific niche indications and are therefore less relevant to the experimental evolution studies discussed here. The eukaryotic nature of fungal pathogens and their hosts accounts for the absence of specialized targets and therefore restricts the efficacy of these antifungal agents. Also, the extensive use of antifungals has created strong selective pressures on pathogenic *Candida* spp., driving the rapid emergence of resistant populations [45,46,47,48]. EE and in vivo studies have been instrumental in providing key insights into how these pathogens acquire and refine mechanisms of drug tolerance, defined as the capacity of a drug-susceptible fungal strain to grow in the presence of antifungal concentrations exceeding the minimal inhibitory concentration, and drug resistance, defined as the ability to grow at drug levels that inhibit susceptible isolates [49]. Importantly, EE approaches often mimic clinically relevant treatment scenarios, such as prolonged drug exposure, stepwise increases in antifungal concentration, or intermittent dosing, reflecting therapeutic regimens used in patients. These designs provide mechanistic insights into clinically observed phenomena, including treatment failure, persistence, and relapse during antifungal therapy [10]. In this context, several EE studies have investigated the adaptive responses of different *Candida* species to distinct antifungal classes, including azoles in *C. albicans* and *C. auris*, echinocandins in *C. glabrata*, and polyenes in multiple *Candida* species. Key experimental evolution studies investigating the mechanisms of antifungal resistance and the resulting genomic adaptations in *Candida* species are summarized in Table 2.

### 6.1. Adaptive Responses to Azole Exposure

Azoles, such as fluconazole, are the first-line antifungals used against fungal infections. They inhibit lanosterol 14α-demethylase (encoded by *ERG11*), a key enzyme in the ergosterol biosynthesis pathway [50]. This inhibition blocks the conversion of lanosterol to ergosterol, leading to the accumulation of toxic sterols such as 14-methyl-3,6-diol, which disrupt membrane integrity and inhibit fungal growth [50]. Fluconazole remains widely prescribed due to its efficacy, safety, bioavailability, and low cost. However, as it is a fungistatic drug, the emergence of resistant isolates is a prevalent problem [51,52]. Known resistance mechanisms include changes in sterol biosynthesis that cause sterols to replace ergosterol; overexpression of the target enzyme; upregulation of drug efflux pumps; and changes in the target gene sequence that cause a reduction in the target protein’s drug binding affinity [53,54,55].

#### 6.1.1. In Vivo Evolution Studies in *Candida albicans*

Among in vivo studies, initial research conducted in the 1990s focused on HIV patients with oral candidiasis resulting from *C. albicans* infection, who received fluconazole treatment over a specific period [56,57,58,59,60,61,62,63]. These studies consistently demonstrated that resistance arises through the overexpression and mutation of key genes, including *ERG11*, *CDR1*, *CDR2*, *MDR1*, *TAC1*, *UPC2*, and *MRR1*, which are now established as canonical mechanisms of azole resistance.

Overexpression of *ERG11* increases the cellular concentration of the azole target enzyme, thereby reducing drug efficacy [57,58,62,63]. This is often linked to mutations in *UPC2*, a Zn(II)_2_Cys_6_ transcription factor that regulates sterol uptake and biosynthesis [64]. For example, the G1927A mutation results in an A643T substitution in Upc2, driving *ERG11* overexpression [65]. Additionally, other studies found that point mutations in *ERG11* itself, resulting in substitutions R467K, D116E, G450E, Y132F, D446N, F126L, K143R, S405F, F449S, T229A, Y132H, and G464S, alter the drug-binding pocket and reduce azole affinity [60,66,67].

Azole exposure also induces overexpression of *CDR1* and *CDR2*, which encode ATP-binding cassette (ABC) efflux pumps [56,58,62,63,66,68]. This overexpression is often linked to the upregulation of *TAC1*, a Zn(II)_2_Cys_6_ binuclear cluster-type transcriptional activator that regulates these and other drug-responsive genes [69]. Gain-of-function mutations in *TAC1*, such as A736V, N977D, and ΔL962–ΔN969, have been associated with increased expression of these efflux pumps [67,70].

A parallel mechanism involves the major facilitator superfamily transporter *MDR1* [71]. Several studies have demonstrated that resistant isolates frequently display elevated *MDR1* mRNA levels [57,58,62,63,68]. These increased levels are driven by the overexpression of *MRR1*, a putative Zn(II)_2_Cys_6_ transcription factor that regulates *MDR1* transcription [72]. Studies showed that gain-of-function mutations such as P683H, P683S, G997V, T896I, Q350L and N803D are responsible for the overexpression of *MRR1* leading to the overexpression of *MDR1* [57,58,66,72,73].

Other studies demonstrated that, in addition to single-gene mutations, structural genomic alterations also play a major role. Coste et al. demonstrated that the isochromosome 5L [i(5L)] increases the expression of *ERG11* and *TAC1*, although the isochromosome is unstable and lost in the absence of fluconazole [67]. Ford et al. identified a trisomy on chromosome V and LOH on chromosome 3R and 5L, which impact efflux and ergosterol biosynthesis genes [74]. Todd et al. further linked LOH breakpoints at long repeats across multiple chromosomes (II, III, V, VI, VII, and R) to genome plasticity in resistant isolates [33].

Collectively, in vivo studies revealed that fluconazole resistance in *C. albicans* is driven by a combination of mechanisms: (i) target alteration via *ERG11* mutations, (ii) efflux activation through *CDR1/CDR2* and *MDR1*, (iii) transcriptional rewiring by *UPC2*, *TAC1*, and *MRR1*, and (iv) large-scale genomic rearrangements that amplify resistance gene dosage. These findings underscore the dynamic and multifactorial nature of resistance evolution, reflecting both gene-specific mutations and genome-wide adaptations under antifungal pressure.

#### 6.1.2. In Vitro Evolution Studies in *Candida albicans*

Although there are several in vivo EE studies, most experiments are carried out in vitro. Cowen and colleagues conducted one of the first experiments, evolving drug-sensitive *C. albicans* from an oral swab of an HIV patient for 330 generations in the presence and absence of fluconazole [75]. At the end of the experiment, populations evolved without fluconazole remained susceptible to fluconazole, itraconazole, ketoconazole, and amphotericin B, whereas those evolved with fluconazole acquired resistance to azoles but retained susceptibility to amphotericin B [75]. Fitness assays revealed that fluconazole-evolved populations had greater fitness in the presence of the drug, but reduced fitness in its absence [76]. Further expression analyses showed that resistance emerged via multiple routes: overexpression of *CDR2* in one lineage, *MDR1* upregulation in several lineages (both early and late microevolution), and alternative mechanisms in others [77].

Using a similar in vitro approach, Selmecki et al. found that fluconazole-evolved strains often carried trisomies of smaller chromosomes (III–VII) and the isochromosome 5L (i(5L)), which also appeared during EE in vivo [78]. Strains harbouring i(5L) displayed enhanced fitness both in the presence and absence of fluconazole, suggesting the existence of compensatory mechanisms that mitigate the fitness costs associated with resistance acquisition [78]. Todd and colleagues further investigated the role of CNV in resistance acquisition. In their evolution experiments with fluconazole, CNVs arose at high frequencies (~1.3 × 10^−6^ to 1.5 × 10^−6^ per gene per cell division), highlighting that fluconazole exposure accelerates CNV [30]. Breakpoints frequently occurred within long inverted repeats, generating dicentric chromosomes that increased genome instability and heterogeneity, both linked to drug tolerance and resistance [30]. Overall, CNVs proved to be rapid, reversible, and capable of conferring cross-resistance to multiple azoles.

Interestingly, when isolates evolved under fluconazole at different concentrations (0, 1, 8, 64 μg/mL) for 100 generations, distinct evolutionary outcomes were observed [79]. While high drug concentrations (8 and 64 μg/mL) selected for drug-tolerant phenotypes without major aneuploidies or LOH events, low drug concentrations (1 μg/mL) conditions favoured the emergence of resistance mutations, including segmental aneuploidies on chromosomes I, III, and IV, formation of i(5L), and LOH on chromosomes V and R [79].

Other evolution studies explored additional mechanisms. Weil and coworkers demonstrated that elevated mistranslation of the CUG codon from serine to leucine in *C. albicans* facilitated fluconazole resistance acquisition through *CDR1*/*CDR2* overexpression driven by a gain-of-function mutation (A736V) in *TAC1*, extensive LOH on chromosome V, and CNV increase across multiple chromosomes [80]. Hill et al. showed that combining fluconazole with inhibitors of *HSP90* (geldanamycin) or calcineurin (F506K) uncovered resistance mechanisms dependent on stress-response regulators. Resistance emerged through point mutations in *HSP90* (G271T) and *CNA1* (S401*), as well as chromosomal aneuploidies affecting chromosomes IV, V, VI, and VII [81].

Huang and colleagues employed Visualizing Evolution in Real-Time (VERT), a novel experimental evolution approach in which cells of the reference strain SC5314 expressed fluorescent proteins (GFP, YFP, or RFP) during chemostat culture. By tracking coloured colonies that increased in abundance under fluconazole exposure, they identified adaptive events appearing within 10–40 generations, which persisted for at least 30 generations even in the absence of the drug [82]. Similarly, Gerstein et al. evolved 20 diverse clinical isolates, each with different resistance and tolerance phenotypes, for 100 generations with fluconazole, showing that strain background strongly influenced evolutionary dynamics. While clade or mating type had no effect, parental genomic background correlated with fitness, tolerance, and genome size variation [83]. Notably, parental strains with MIC values above 1 μg/mL exhibited slow growth of a subpopulation of cells at high drug concentrations, leading to increased tolerance and greater diversity in genome sizes. Furthermore, parental strains with low fitness were associated with high levels of heterogeneity in fitness, tolerance, and genome size during evolution [83].

Other azoles, such as posaconazole, have also been investigated. Kukurudz et al. performed an EE study using multiple replicates of seven clinical *C. albicans* isolates and the reference strain SC5314, all exposed to high concentrations of the drug [84]. The study demonstrated that the emergence of stable drug resistance to posaconazole was rare; however, increased drug tolerance was frequently observed across strains. Moreover, whole-genome sequencing revealed that 11 of 12 evolved SC5314 replicates exhibited trisomy of at least one of chromosomes III, VI, or R, suggesting a potential genetic adaptation underlying tolerance to posaconazole [84].

#### 6.1.3. Experimental Evolution Studies of Other *Candida* Species

Beyond *C. albicans*, EE studies have shed light on resistance mechanisms in other pathogenic *Candida* species. In *C. glabrata*, in vivo evolution was documented in two AIDS patients with oropharyngeal candidiasis treated with fluconazole, where clinical isolates acquired resistance through *CDR1* overexpression [85]. This overexpression was caused by the hyperactivity of the transcription factor *PDR1* through gain-of-function mutations (R376W, D1026, T588A, E1083Q, Y584C, L280F, P822L) [86]. These *PDR1* mutations not only conferred resistance but also enhanced virulence, as demonstrated by fluconazole treatment failure in a mouse model [87]. This effect is likely due to decreased macrophage adherence and uptake, coupled with increased epithelial adherence via upregulation of the adhesin encoding gene *EAP1* [87,88].

In vitro evolution studies of *C. glabrata* confirmed this pattern. Cavalheiro and colleagues conducted a transcriptomic analysis of a *C. glabrata* clinical isolate that transitioned from azole susceptibility to resistance against posaconazole, clotrimazole, fluconazole, and voriconazole following long-term fluconazole exposure [89]. Only the population that acquired resistance to all azoles carried a gain-of-function mutation in *PDR1* (Y372C), which resulted in the overexpression of multidrug resistance transporter genes [89]. In contrast, strains exhibiting resistance to posaconazole and clotrimazole, along with elevated MIC values for fluconazole and voriconazole, were found to rely on alternative resistance mechanisms, including adhesin gene upregulation and biofilm-related mechanisms, such as *EPA3*-mediated reduction in intracellular drug accumulation [89]. *C. glabrata* fluconazole-induced resistance has also been associated with duplications of chromosomes A and E, as well as point mutations in *ERG11* within the azole-binding pocket (K152, Y141) [90]. Furthermore, Galocha et al. demonstrated a novel mechanism in which loss-of-function mutations in hexose transporters (*CgHXT4/6/7*) reduced azole import, leading to resistance independently of *PDR1* or *ERG11* alterations [91].

In the emerging pathogen *C. auris*, in vitro evolution studies revealed rapid acquisition of azole resistance, primarily through aneuploidies of chromosome V leading to overexpression of *TAC1B, NCP1, ERG9,* and *ERG13* [92,93,94]. Point mutations in *TAC1B* (R495G, F214S, FS191S, V742A) were also common and associated with increased *CDR1* expression [95,96]. Additional mechanisms include *UPC2* mutations (C444Y, A506V), overexpression of ergosterol biosynthesis genes (*ERG1*, *ERG7*, *ERG8*, *ERG11*, *ERG26*), efflux pump upregulation, and aneuploidies affecting chromosomes I, III, and VI [92,93,94,95,96].

Overall, experimental evolution studies under azole selection demonstrate that *Candida* species exhibit remarkable adaptability to these antifungals through a diverse and multifactorial set of mechanisms. The trajectory of resistance is highly context-dependent, influenced by genetic background, ploidy, drug concentration, and environmental conditions, with resistance and tolerance emerging as distinct but interacting outcomes. Importantly, these adaptive processes are observed both in vivo, mimicking clinical treatment conditions, and in vitro under controlled experimental evolution, highlighting the predictive value of EE for understanding antifungal adaptation.

Clinically, these findings emphasize the need to consider both genetic and environmental variables when designing antifungal strategies. The rapid and repeatable emergence of resistance underscores the limitations of monotherapy and the importance of monitoring evolutionary trajectories during treatment, particularly in immunocompromised patients or in the presence of microbiome-altering interventions.

### 6.2. Evolutionary Responses to Echinocandin Exposure

Echinocandins are lipopeptides derived from pneumocandins that inhibit the 1,3-β-D-glucan synthase enzyme, composed of the intracellular regulatory subunit Rho1 and the transmembrane catalytic subunits Fks [97]. By targeting the Fks1 subunit, echinocandins block the conversion of glucose uridine diphosphate into 1,3-β-D-glucan, a key cell wall polymer. The resulting impairment of cell wall synthesis causes osmotic instability and ultimately cell death [97]. Resistance arises through mutations in specific regions (“hotspots”) of the *FKS1* gene, which reduce the affinity of the drug for its target enzyme [98].

EE studies with echinocandins in *C. albicans* are scarce. However, one study demonstrated that, under caspofungin treatment, tetraploid strains adapted more rapidly and reached higher resistance levels than diploid strains [99]. Tetraploid populations underwent rapid genome-size reductions prior to acquiring resistance, whereas diploids generally maintained stable genome sizes. Resistance was linked to distinct types of *FKS1* mutations, and aneuploidies on chromosome II and V. Furthermore, tetraploid-evolved lines exhibited lower fitness costs in the absence of drug pressure. Collectively, these findings suggest that the transient tetraploid state enhances adaptability and may contribute to the emergence of antifungal drug resistance [99].

Most research in this area has been carried out with other species, particularly *C. glabrata*. In in vivo evolution, Singh-Babak and colleagues analyzed clinical isolates of *C. glabrata* from a patient with recurrent bloodstream candidemia treated with caspofungin over 10 months [100]. They identified missense mutations in multiple genes, including *MOH1* (Y5H), *GPH1* (H409Y), *TCB1/2* (A1161V), *DOT6* (K347*), *MRPL11* (Y161H), *SUI2* (I121M), *FSK2* (S663P), *CDC6* (K171E), and *CDC55* (P155S). Of these, only two were functionally validated: *CDC6*-A511G(K171E), which conferred modest resistance, and *FKS2*-T1987C(S663P) which was the primary driver of echinocandin resistance. The *FSK2* substitution reduced fitness, but this cost was compensated by *CDC55*-C463T(P155S) that resulted in *CDC55* overexpression. Moreover, *FKS2*-T1987C(S663P)-mediated resistance was dependent on Hsp90 and calcineurin signalling [100]. Additional missense mutations in *FSK2*, including D66H, L664R, D666E, D66N, and ΔF659, have also been identified in resistant clinical isolates of *C. glabrata* [101].

In in vitro EE, resistance has been documented in *C. glabrata* [90,101,102,103,104], *C. parapsilosis* [104], and *C. auris* [94,105]. Across these species, resistance emerged through mutations in *FKS1* or *FKS2*, either within or outside hotspot regions of the genes encoding 1,3-β-glucan synthase. In *C. glabrata*, *FKS1* resistance was linked to mutations outside hotspots (R1422L, F708S, W681L) [90], while *FKS2* resistance involved hotspot mutations (S663P, ΔF658, W715L) [101,102,103] and non-hotspot alterations (K265*, A651T, ΔF659) [90]. In *C. parapsilosis*, resistance was conferred by a heterozygous hotspot 1 mutation (*FKS1* S656P) and homozygous mutations in hotspot 2 (*FKS1* W1370R) and hotspot 3 (*FKS1* L703F) [104]. In *C. auris*, resistance was associated with a codon deletion in hotspot 1 (*FKS1* FL635L) [94]. Additional resistance mechanisms include a truncation in *CEN1* and duplication of chromosome L in *C. glabrata* [90], as well as an *ERG3* L207I substitution in *C. auris* [94].

In summary, EE studies with echinocandins remain scarce in *C. albicans*, but work in *C. glabrata*, *C. parapsilosis*, and *C. auris* has shown that resistance is largely driven by mutations in *FKS1* and *FKS2*. These mutations, whether inside or outside hotspot regions, reduce drug binding affinity to glucan synthase. Other genomic alterations, such as gene truncations, chromosomal duplications, and mutations in sterol biosynthesis genes, further contribute to resistance. These studies highlight that echinocandin resistance, although less frequent than azole resistance, can emerge rapidly under sustained drug pressure and may involve fitness trade-offs that are subsequently compensated. The strong dependence of resistance on specific *FKS* mutations supports their use as molecular markers for surveillance and diagnostics.

### 6.3. Adaptive Mechanisms Under Polyene Evolutionary Pressure

Polyenes, such as amphotericin B, act through a unique mechanism: instead of targeting an enzyme, they directly bind to ergosterol, creating pores in the fungal membrane. Resistance is less common but can emerge through alterations in sterol composition, typically through mutations in genes of the ergosterol biosynthesis pathway [106].

**Table 2:** Summary of adaptive mechanisms and genetic drivers of antifungal resistance in Candida species identified through EE.

Antifungal Class	Primary Target	Species	Evolution Setting	Key Resistance Mechanisms	Associated Genes & Genomic Alterations
Azoles	Lanosterol 14α-demethylase (ERG11)	*C. albicans*	In vivo (HIV patients) [56,57,58,59,60,61,62,63] & in vitro [75,76,77,78,79,80,81,82,83,84]	Target modification and overexpression [57,58,59,60,61,62,63,64,65,66,67]; increased drug efflux [56,57,58,59,60,61,62,63,64,65,66,67,68,69,70,71,72,73]; transcriptional rewiring [69,70,71,72,80]; stress-response dependence [81]	Genes: *ERG11*, *CDR1*, *CDR2*, *MDR1* [56,57,58,59,60,61,62,63,64,65,66,67,68] TFs: *TAC1*, *UPC2*, *MRR1* [65,69,70,71,72,73,80] Genomic: i(5L), Trisomy (Chr III–VII, R), LOH (Chr 3R, 5L, V, R) [30,33,67,74,78,79,80,84]
		*C. glabrata*	In vivo (AIDS patients) [85,86,87] & in vitro [89,90,91]	Transcription factor hyperactivity [86,89]; altered drug import [91]; biofilm and adhesion-mediated mechanisms [87,88,89]	Genes: *PDR1* (GOF mutations) [86,89], *ERG11* [90], *CgHXT4/6/7* [91], *EPA3*, *EAP1* [87,88,89] Genomic: Duplication of Chromosomes A and E [90]
		*C. auris*	In vitro [92,93,94,95,96]	Rapid acquisition via aneuploidy; target and efflux pump upregulation [92,93,94,95,96]	Genes: *TAC1B*, *CDR1*, *UPC2*, *ERG1*, *ERG7*, *ERG8*, *ERG11*, *ERG26*, *NCP1* [92,93,94,95,96] Genomic: Aneuploidies of Chr V (common), I, III, and VI [92,93,94]
Echinocandins	1,3-β-D-glucan synthase (FKS subunits)	*C. albicans*	In vitro [99]	Rapid adaptation in tetraploid strains; genome-size reduction; target site mutations [99]	Genes: *FKS1* [99] Genomic: Aneuploidies in Chr II and V [99]
		*C. glabrata*	In vivo [100] & in vitro [90,101,102,103]	Target modification (hotspot and non-hotspot) [90,100,101,102,103]; stress-signalling dependence (Hsp90/calcineurin) [100]	Genes: *FKS1*, *FKS2* (primary driver) [90,100,101,102,103], *CDC6*, *CDC55* (compensation) [100], *MOH1*, *GPH1*, *TCB1/2* [100] Genomic: Duplication of Chr L; CEN1 truncation [90]
		*C. auris*	In vitro [94,105]	Point mutations and codon deletions in FKS hotspots [94,104]	Genes: *FKS1* (hotspot 1), *ERG3* [94]
		*C. parapsilosis*	In vitro [104]	Point mutations in FKS hotspots [104]	Genes: *FKS1* (hotspots 1, 2, 3) [104]
Polyenes	Ergosterol (pore formation)	*C. albicans*	In vitro & in vivo (mouse model) [107]	Alterations in sterol composition; loss of virulence and fitness; Hsp90 dependence [107]	Genes: *ERG6* (point mutations) [107] Genomic: LOH [107]
		*C. auris*	In vitro [94]	Nonsense mutations in ergosterol pathway; DNA damage checkpoint alterations [94]	Genes: *ERG3*, *ERG11*, *MEC3* [94]

Abbreviations: LOH, Loss of Heterozygosity; i(5L), Isochromosome 5L; Chr, Chromosome.

In *C. albicans*, Vicent and colleagues investigated the rare mechanisms of amphotericin B resistance, focusing on LOH events in genes of the ergosterol biosynthesis pathway [107]. During in vitro evolution with amphotericin B in the laboratory strain *C. albicans* ATCC-10231, resistance emerged through point mutations and LOH in *ERG6* [107]. These mutations were dependent on Hsp90 activity for cell survival and were associated with defective filamentation [107]. Importantly, in a mouse model, amphotericin B–resistant *C. albicans* strains were avirulent. The mutations impaired the ability of *Candida* to withstand host-associated stresses, crippled filamentation (a major virulence factor), and abolished lethality in mice [107].

In *C. auris*, in vitro EE revealed additional resistance mechanisms. Resistance to amphotericin B arose through nonsense mutations in *ERG3* (W182*) and *ERG11* (E429*), as well as a missense mutation in *MEC3* (A272V), a gene involved in DNA damage checkpoint signalling [94]. The *ERG3* and *ERG11* mutations also conferred cross-resistance to fluconazole, further complicating treatment [94].

Overall, EE studies have shown that amphotericin B resistance in *Candida* species is rare and often incurs substantial fitness and virulence costs. These findings highlight a pronounced evolutionary trade-off between resistance and pathogenicity. Amphotericin B–resistant strains often show reduced fitness, attenuated virulence, and diminished ability to cope with host-associated stresses, which may limit their persistence in clinical settings. This helps explain the continued clinical durability of polyenes despite decades of use.

## 7. Conclusions and Future Perspectives

EE has proven to be a powerful tool for investigating how *Candida* species adapt to diverse selective pressures, from host-imposed stresses to antifungal drugs. By allowing real-time observation of adaptive trajectories, this approach has revealed the remarkable genomic plasticity of *Candida* and its ability to exploit multiple evolutionary routes towards survival. These studies have deepened our understanding of the mechanisms underpinning antifungal resistance, the role of chromosomal rearrangements and aneuploidy in adaptation, and the trade-offs between fitness, virulence, and drug resistance.

Despite these advances, important challenges remain. Most EE studies have focused on a limited set of species (*C. albicans* and *C. glabrata*), while emerging pathogens such as *C. auris* and *C. parapsilosis* are underrepresented. Furthermore, most studies have concentrated on azoles, leaving echinocandins and polyenes, the other two major antifungal classes, relatively underexplored, despite their frontline clinical use. Another notable gap is the lack of EE studies involving drug combinations or sequential therapies, even though these approaches are increasingly used in clinical settings to manage challenging infections.

A particularly critical area for future work is the expansion of in vivo EE studies. While in vitro models provide valuable insights under controlled conditions, they cannot fully replicate the complex and fluctuating selective pressures encountered within the host. In vivo studies capture interactions with the immune system, nutrient limitation, tissue-specific environments, and drug pharmacodynamics, all of which strongly influence evolutionary trajectories. Another key frontier lies in understanding the interplay between resistance and virulence. Evidence suggests that resistance mutations often impose fitness costs or attenuate pathogenic traits, yet compensatory mechanisms can restore fitness and maintain virulence. Dissecting these evolutionary trade-offs will be crucial for predicting the trajectories of resistant strains in clinical contexts. Thus, in vivo EE is essential for understanding the clinical relevance of resistance pathways and for predicting how *Candida* adapts during infections. Priority questions for future work include how specific immune pressures shape resistance pathways, how drug penetration in different tissues constrains evolution, and whether resistance–virulence trade-offs observed in vitro persist during infection. Addressing these questions will require standardized animal models, longitudinal sampling, and integration of genomic and phenotypic data across time.

Looking forward, the field would benefit from broadening EE to encompass a wider diversity of *Candida* species, antifungal classes, and therapeutic strategies, with a deliberate integration of in vivo models. Key directions include systematic EE of underrepresented species such as *C. auris* and *C. parapsilosis*; experimental testing of combination and sequential therapies; and direct comparison of resistance trajectories across antifungal classes. When coupled with advanced approaches such as long-read sequencing, single-cell omics, CRISPR-based genome editing, evolutionary modelling, and machine learning, EE can generate mechanistic and predictive insights directly applicable to the clinical practice. These integrative EE strategies can inform antifungal stewardship by identifying treatment regimens that minimize resistance emergence, and guide drug discovery by revealing evolutionarily constrained targets. This will support personalized antifungal therapy by anticipating patient-specific evolutionary trajectories.

## Data Availability

No new data were created or analyzed in this study. Data sharing is not applicable to this article.

## Abbreviations

The following abbreviations are used in this manuscript:

EE	Experimental evolution
LOH	Loss of heterozygosity
CNV	Copy number variation
NAC	Non-*albicans Candida*
SDC	Synthetic defined medium
MM	Minimal Medium
Pdep	Phosphorus-depletion medium
Ndep	Nitrogen-depletion medium
SNP	Single-Nucleotide Polymorphism
INDEL	Insertion and Deletions
ROS	Reactive oxygen species
AMPs	Antimicrobial peptides
ABC	ATP-binding cassette
VERT	Visualizing Evolution in Real-Time
